# TGF-β signaling promotes cervical cancer metastasis via CDR1as

**DOI:** 10.1186/s12943-023-01743-9

**Published:** 2023-03-31

**Authors:** Guanglei Zhong, Qian Zhao, Zhiliao Chen, Tingting Yao

**Affiliations:** 1 Department of Gynecological Oncology, Sun Yat-sen Memorial Hospital, Sun Yat-sen University, 107 Yan Jiang West Road, Guangzhou, People’s Republic of China 510120; 2 Guangdong Provincial Key Laboratory of Malignant Tumor Epigenetics and Gene Regulation, Sun Yat-Sen Memorial Hospital, Sun Yat-Sen University, Guangzhou, 510120 China

**Keywords:** Cervical cancer, Metastasis, TGF-β, CDR1as, *Slug*, IGF2BP1

## Abstract

**Background:**

Due to the lack of effective treatment, metastasis is the main cause of cancer related deaths. TGF-β pathway has been reported related to cervical cancer metastasis. However, mechanism is still unclear.

**Methods:**

After agonist of TGF-β treatment, RNA sequencing revealed the expression profiles of circRNA in cervical cancer. In situ hybridization was used to analysis relationship between CDR1as and prognosis. Real-time PCR, Western blot, RNA interference, Transwell assay, Wound healing assay, RNA pulldown assay and RIP assays were performed in vitro. And in vivo cervical cancer model (including foot pad model and subcutaneous tumor formation) was also performed.

**Results:**

CDR1as was found upregulated obviously following TGF-β activation. In situ hybridization showed CDR1as was positively correlated with lymph node metastasis and shortened survival length. Simultaneously, overexpression of CDR1as promoted cervical cancer metastasis in vitro and in vivo. It was also found that CDR1as could facilitate the orchestration of IGF2BP1 on the mRNA of SLUG and stabilize it from degradation. Silencing IGF2BP1 hampers CDR1as related metastasis in cervical cancer. Additionally, effective CDR1as has been proven to activate TGF-β signaling factors known to promote EMT, including P-Smad2 and P-Smad3.

**Conclusions:**

Our study proved TGF-β signaling may promote cervical cancer metastasis via CDR1as.

## Introduction

Although cervical cancer screening and administration of HPV vaccines have been widely used, cervical cancer still remains the fourth greatest global burden of female cancer on incidence and mortality because of metastasis [1–3]. The overall survival rate for metastasis is still poor. Hence, effective predictive biomarkers are vital to alleviate the progression.

More and more evidence show that metastasis is a multi-step process involving complex interaction of transcriptome changes. TGF-β is a multifunctional cytokine that is associated with cancer metastasis in various cancer cells [4]. While it is well known that TGF-β-induced metastasis is very complex, the underlying mechanisms involved in cervical cancer are not fully understood.

N6-methyladenosine (m6A) is the most common modification in messenger RNA (mRNA). The insulin-like growth factor 2 mRNA-binding proteins (IGF2BPs) have been considered as m^6^A readers, which could target thousands of mRNA transcripts through recognizing the consensus GG(m^6^A) C sequence [5]. IGF2BPs belong to a conserved family of RNA-binding oncofetal proteins. Several studies have shown that IGF2BPs could influence polarization, migration, morphology, metabolism, proliferation and differentiation [6–8].

CDR1as is a conventional circular RNA with a length of 1485 bp. It is located in chromosome X and directly transcribed from LINC000632 [9, 10]. There are 73 binding sites between CDR1as and let-7. It was found to promote brain maturation [11] and accelerate lung [12–14], esophagus [15, 16], and bladder cancer progression [17, 18]. Additionally, it has also been proven to be involved in cancer drug resistance [19]. The effects were suggested on a multi-post-transcriptional level [10, 20]. So, it seems that CDR1as may function as a potential biomarker and therapeutic target. However, the role of CDR1as in cervical cancer has not been clearly identified.

In this study, we firstly induced endothelial-mesenchymal transition (EMT) in cervical cancer via TGF-β induction; then, the expression of different circular RNAs was analyzed. It was found that CDR1as was significantly elevated when EMT was induced and stabilized Slug expression via binding with IGF2BP1. Our research firstly addressed that TGF-β played a major part in the regulation of CDR1as and required m6A modification on multiple metastatic-related genes in cervical cancer.

## Material and methods

### Patient information

65 patients diagnosed with cervical cancer from July 2010 to February 2014 in Sun Yat-sen University were recruited. All patients signed the contract for permission to use their tissue for experimental purposes. All tissues that were used for pathological classification were assessed by at least three pathologists. Chips were purchased from Shanghai Outdo Biotech Co., Ltd., including 111 cervical cancer tissue samples.

### Cell culture and reagents

Hela, Siha, and C33a were all cultured in DMEM (HyClone, USA) culture medium, with 10% FBS (Gibco, USA), 1% penicillin streptomycin (Gibco, USA), 1% Mycoplasma Removal Agent (Yeasen Biotechnology, Shanghai), and were stored at 37 °C and a humidified atmosphere with 5% CO_2_ (Thermo Fisher, Germany). The TGF-beta activator SRI-011381 hydrochloride and the TGF-beta inhibitor LY2109761 were purchased from Selleck Chemicals.

### RNase R digestion

RNA (1 μg) was digested with 3 μL 10X RNase R reaction buffer and 3 U RNase R for 2 h at 37 °C. 500 μL pre-cooled isopropanol, 1 μL glycogen, and 5 μL 30% NaAc were added into each sample. RNA was precipitated at− 80 °C for 24 h and extracted.

### Agarose electrophoresis

Cell genomic DNA was extracted using human genomic DNA extraction kit (Kangwei, China). Cell cDNA was reverse transcribed using reverse transcription kit (TaKaRa, Japan). Primers for CDR1, CDR1as, and linc were concluded in Table 1. 2% agarose was diluted with boiled 0.5% TAE and cooled for gel making. 9 μL of cDNA and gDNA, along with 1 μL of loading buffer were added to each hole for electrophoresis. Gels were luminated by Invitrogen iBright CL1000.

**Table 1:** Primers

Primer	sense (5′-3′)	Anti-sense(5′-3′)
CDR1as	TCTGCTCGTCTTCCAACATC	CGGAAACCCTGGATATTGCA
CDR1	CGGATTTCCTGGAAGACCTGGA	TCCGTGTCTTCCAGCAAGTCCA
β-actin	CACCATTGGCAATGAGCGGTTC	AGGTCTTTGCGGATGTCCACGT
GAPDH	GTCTCCTCTGACTTCAACAGCG	ACCACCCTGTTGCTGTAGCCAA
MALAT1	TTCCGGGTGTTGTAGGTTTC	AAAAAACCCACAAACTTGCC
E-cadherin	GCCTCCTGAAAAGAGAGTGGAAG	TGGCAGTGTCTCTCCAAATCCG
N-cadherin	CCTCCAGAGTTTACTGCCATGAC	GTAGGATCTCCGCCACTGATTC
SLUG	ATCTGCGGCAAGGCGTTTTCCA	GAGCCCTCAGATTTGACCTGTC
SNAIL	TGCCCTCAAGATGCACATCCGA	GGGACAGGAGAAGGGCTTCTC
SMAD2	GGGTTTTGAAGCCGTCTATCAGC	CCAACCACTGTAGAGGTCCATTC
SMAD3	TGAGGCTGTCTACCAGTTGACC	GTGAGGACCTTGTCAAGCCACT
TWIST	GCCAGGTACATCGACTTCCTCT	TCCATCCTCCAGACCGAGAAGG
VIM	AGGCAAAGCAGGAGTCCACTGA	ATCTGGCGTTCCAGGGACTCAT
ZEB1	GGCATACACCTACTCAACTACGG	TGGGCGGTGTAGAATCAGAGTC
ZEB2	AATGCACAGAGTGTGGCAAGGC	CTGCTGATGTGCGAACTGTAGG
IGF2BP1	CTTTGTAGGGCGTCTCATTGGC	CCTTCACAGTGATGGTCCTCTC
IGF2BP2	GTTGGTGCCATCATCGGAAAGG	TGGATGGTGACAGGCTTCTCTG
IGF2BP3	TCGTGACCAGACACCTGATGAG	GGTGCTGCTTTACCTGAGTCAG
SLUG site1	TAAAGGAGCCGGGTGACTTC	TGTATGTGTGTCCAGTTCGC
SLUG site2	AAGCCAAACTACAGCGAACT	AACAGTTGAATCTTTGGCTCTTT
SLUG site3	TGCCTGTCATACCACAACCA	GAGGTGTCAGATGGAGGAGG
SLUG site4	ACTACCGCTGCTCCATTCC	GGGTCTGAAAGCTTGGACTG
SLUG site5	GCGCCCTGAAGATGCATATT	TCATGCAAATCCAACAGCCA
SLUG site6	CAGACCCTGGTTGCTTCAAG	AGCAAGAAATGGAGCACTTTGT
SLUG site7	GTGCTTTTAATGATGGACAGTCA	ACAGAACACACATTCAAGCACA

### CircRNA expression profiles in Cervical cancer cell

After treatment of SRI-011381 hydrochloride for three days, Human circRNA Arrays (8 × 15 K, Arraystar, Rockville, MD, USA) was used to detect the different circRNAs with ≥ 2 folds. The data were analyzed by using R software limma and Arraystar program (Arraystar).

### Transient transfections and RNA interference

Cells with 30–50% density in 6 well plates were plated 24 h before transfection. Lipofectamine iMAX mixed with siRNAs in opti-MEM was added to each plate. RNA, protein, and cells were harvested for later research after 48 h transfection. siRNA sequences for each target were concluded in Table 1.

### Transwell assay

Eight thousand cells were counted and suspended in 200 μL non-serum culture medium (for metastasis assay) or 200 μL non-serum matrix gel (for invasive assay) in upper-channel, while the lower channel was added with 600 μL complete medium. After 24 h, cells were fixed with 500 μL paraformaldehyde and stained with crystal violet. Cells that penetrated through the membrane were observed and photographed with an optical microscope. The relative rate for metastasis and invasion was analyzed and compared with the control group.

### Wound-healing assay

Cells with 100% density were plated in 24-well plates. After 24 h, 1 mL pipette was used to create the indicated cellular wound. To observe the healing of wounds, cells were photographed microscopically after 0 h, 12 h, 24 h and 48h from the time of wound creation.

### In situ hybridization in tissue (ISH)

Tissues that were embedded in paraffin were individually sliced. Detailed procedures were strictly followed using the enhanced sensitive ISH Detection Kit I (MK1030). Probes of CDR1as were purchased from GeneBio, Shanghai, China. The sequence was 5′ > T + GC + CATCCGGAAAC+C + CTGGATATTGCAGACACTGGAAGACC+TGA + AT< 3′. The expression strength was scored from 0 to 9, and the strength was calculated by multiplication between staining area and intensity. The staining area was defined as such: grade 0: ≤25% positive cells; grade 1: ≤50% positive cells; grade 2: ≤75% positive cells; grade 3: ≤100% positive cells. The staining intensity was defined as such: grade 0: no staining color; grade 1: yellow stain; grade 2: pale brown stain; grade 3: deep brown stain.

### In situ florescence hybridization

Ten thousand cells were plated in confocal dishes for 24 h, then fixed with 4% paraformaldehyde. The exact procedures were strictly adhered to using the Fluorescence In Situ Hybridization Kit (RIBOBIO, Guangzhou, China). Positive cells were photographed with a Laser Scanning Confocal Microscope, which was magnified 400-fold. Each sample was randomly photographed more than 10 times. Probes of CDR1as were purchased from Synbio Technologies, and the sequence is GGT + GCCAT+CGGAAACCCT+ GGAT+ ATT+ GCAGACA.

### In vivo tumor model

Nude mice (4–6-week-old female mice) were purchased from the animal center of Sun Yat-sen University. Procedures strictly followed the ethical regulations of animal experimentation in Sun Yat-sen University. Subcutaneous tumor formation: 1,000,000 Siha cells were subcutaneously injected at the axillary region of nude mice. Foot pad model: 2,000,000 Siha cells were subcutaneously injected at the foot pad of nude mice. Tumors were observed and measured once a week. Volume was calculated by the function of (V = (L*W^2^)/2) (V: tumor volume; L: tumor length; W: tumor width). Tumors were harvested from the mice when volume reached 1000 mm^3^.

### RNA-pulldown assay

To pulldown proteins with CDR1as, biotin-labeled probes were designed and synthesized (GenePharma, Suzhou, China). 50 μL streptomycin magnetic beads were incubated with CDR1as probe and oligo probe at room temperature for 2 h. At the same time, 10^7^ cells were fixed and lysed. Cell lysates were mixed with probe-coated beads and rotated overnight at 4 °C. Samples were separated by a magnetic separator and washed with pre-coated PBS for 3 times. 50 μL loading buffer was added and beads were boiled for 5 min. Then, the exact expression of indicated protein was examined by Western blot.

### RNA binding protein immunoprecipitation assay (RIP)

Detailed processes during this section were performed according to the recommendation of Magna RIP Kit (MILLIPORE, Germany. Catalog No. 17–700). Antibody for m6A was purchased from ABCAM; IGF2BPs from SANTA.

### Protein extraction and Western blot

Harvested cells were washed with PBS and lysed with RIPA (with 1% protease inhibitor and 1% phosphatase inhibitor). Protein content was measured by BCA assay (Thermo Fisher, Germany). Antibody for SMAD2 (Cat.5339), SMAD3 (Cat.9523), p-SMAD2 (Cat.5339), p-SMAD3 (Cat.9520), Vimentin (Cat.46173), E-Cadherin (Cat.14472), N-Cadherin (Cat.13116), and β-actin (Cat.3700) were purchased from CST (U.S.A). Slug (Cat.166476), IGF2BP1 (Cat.166344), IGF2BP2 (Cat.377014), and IGF2BP3 (Cat.365640) were purchased from SANTA(U.S.A).

### RNA extraction and real-time PCR

Harvested cells were washed with PBS and lysed with Trizol (TaKaRa, Japan). Detailed procedures were followed form the instruction of TaKaRa. RNA was reverse-transcribed by PrimeScriptTM RT Master Mix Kit (TaKaRa, Japan). TB Green Premix Ex Taq II Kit (TakaRa, Japan) was used for quantitative real-time PCR and analyzed by Light Cycler 480 (Roche, Germany).

### Statistical analysis

Statistical analysis was performed by using SPSS 20.0 and GraphPad 8.0. T test was used between two individual groups. Chi-squared test was used for correlation analysis. One-way ANOVA analysis were used among multiple groups. Survival rate of patients was evaluated by Kaplan-Meier curves and log-rank tests. *P* < 0.05 was considered as statistically significant.

## Results

### CDR1as was significantly upregulated after induction of TGF-β in cervical cancer cells

**Fig. 1:**
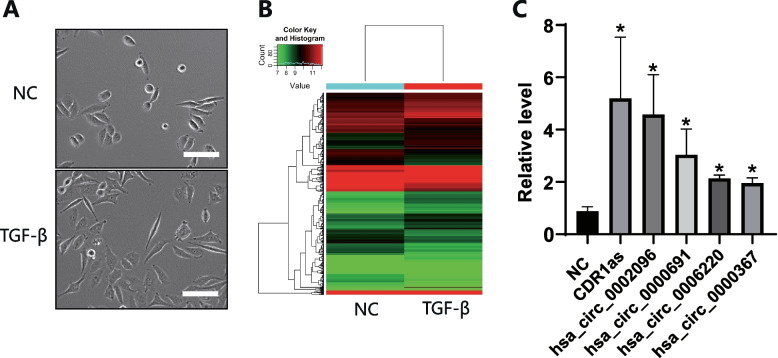
Induction of EMT by TGF- β in cervical cancer cells. **A** Morphological changes of EMT in cervical cancer cells were induced by SRI-011381 treatment. **B** The cluster heat map demonstrated the differentially expressed circRNAs. **C** Real time PCR was used to verify the differential circRNAs expression, and the top five circRNAs with the most obvious upregulation were shown in the figure

EMT is one of the hallmarks in the early stage of tumor progression, and its signal maintenance depends heavily on TGF- β activation. EMT phenotype was induced in Siha cells after treatment with SRI-011381 for 3 days (Fig. 1A). RNA Sequencing showed expression of 6964 circRNAs was up-regulated and 6596 down- regulated (Fig. 1B). We chose 50 circRNAs with the most obvious changes for RT-PCR test. The results showed that the fold change of CDR1as was the highest (Fig. 1C). Therefore, we boldly speculated that CDR1as was involved in cervical cancer metastasis induced by the TGF-β pathway.

### CDR1as predicted lymph node metastasis and worse overall survival

**Fig. 2:**
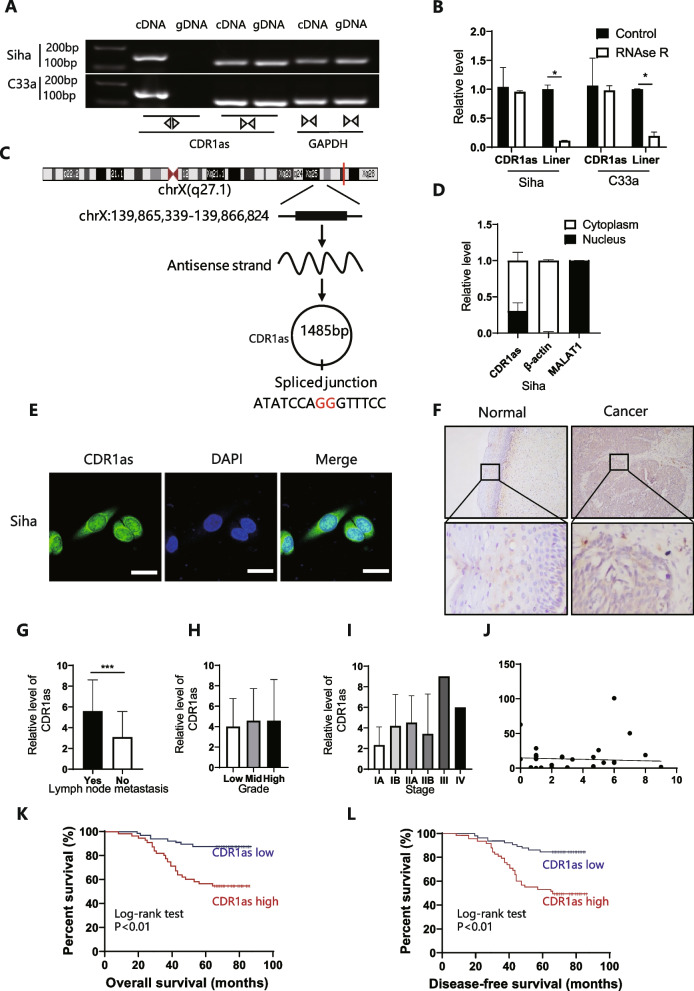
Higher CDR1as level indicated lymph node metastasis and worse overall survival of patients with cervical cancer. **A** To detect if CDR1as is a circular RNA, divergent and convergent primers were designed, agarose gel electrophoresis showed that CDR1as was amplified by divergent primer in cDNA but not in gDNA. GAPDH was taken as the negative control; **B **RNase R treatment assay was performed, and only CDR1as showed strong stability but not its linear origin; **C** Chromosome location and transcripts of CDR1as; **D**,** E **Nuclear-plasma extraction assay and FISH showed that CDR1as was mainly expressed in cytoplasm; **F **ISH was used to evaluate the expression level of CDR1as in cervical cancer tissue; **G**, **H**, **I**,** J** CDR1as level among cervical cancer tissue was analyzed by lymph node metastasis, tumor FIGO stage, tumor grade, and tumor size; *P < 0.05, **P < 0.01; ns, not significant; unpaired Student’s t test **K**,** L** K-M analysis of CDR1as in 111 cervical cancer tissue samples

RNase R digestion assay revealed that the circular form of CDR1as could only be amplified in cDNA sample, while with CDR1, the line form existed in both gDNA and cDNA (Fig. 2A); after being digested by RNase R, the circular form remained significantly more stable compared with its line form (Fig. 2B). Sanger sequencing also indicated that the sequence of amplified oligo was similar to the sequence in www.circbase.com (Fig. 2C). These results demonstrated that CDR1as was a circular RNA.

FISH and nuclear-plasma extraction assay indicated that CDR1as was mainly expressed in the cytoplasm (Fig. 2D, E), which suggested that CDR1as may work post-transcriptionally. In situ hybridization was used to evaluate the relationship between CDR1as and clinical parameters (Fig. 2F, Table 2), which demonstrated a higher expression of CDR1as among lymph node metastasis (Fig. 2G) and correlation with shorter overall survival and worse prognosis (Fig. 2K, L). However, there was no significant relationship between CDR1as and tumor grade, stage and primary tumor volume (Fig. 2H-J).

**Table 2:** CDR1as with clinicopathological factors

	Low	High	*χ2*	*P*-value
Age (years)				
≤ 45	13	15	0.371	0.543
>45	20	17		
Figo stage				
I	32	29	1.132	0.355
≥ II	1	3		
Lymph node metastasis				
No	26	11	13.069	< 0.001
Yes	7	21		
Grade				
Low	14	12	0.167	0.92
Mid	17	18		
High	2	2		
Metastasis				
No	32	29	1.132	0.287
Yes	1	3		

### CDR1as may promote metastasis of cervical cancer cells

**Fig. 3:**
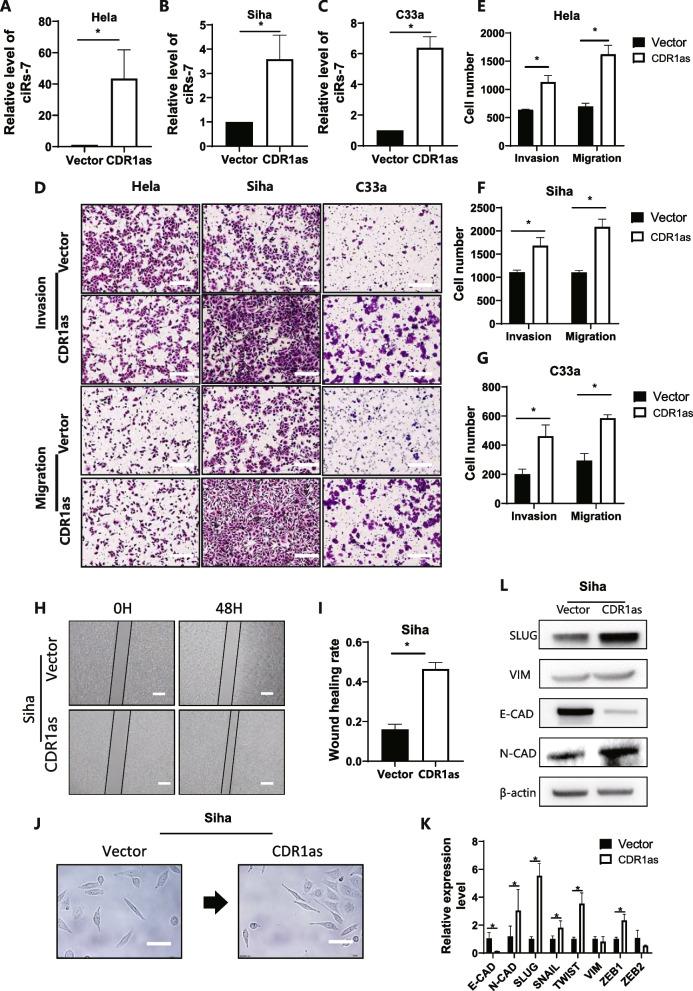
CDR1as promoted metastasis of cervical cancer cells. **A**,** B**,** C** CDR1as level was detected by qPCR after overexpression of CDR1as; **D**,** E**,** F**,** G** Transwell assay was performed to evaluate the migration and invasion ability after overexpression of CDR1as; **H**,** I **Wound healing assay was performed after overexpression of CDR1as; **J **Morphology change after CDR1as was overexpressed in cervical cancer cells; **K**,** L** qPCR and Western blot demonstrated an obvious change of metastasis-related genes and proteins

After overexpression of CDR1as in cancer cells (Fig. 3A-C), metastasis of cervical cancer was significantly expedited, according to more penetrated cells in the membrane from the Transwell assay (Fig. 3D), and shorter wound healing time (Fig. 3H). As illustrated, the motility of cancer cells was enhanced by increase of the invasive and metastatic rate by 1.86-fold and 2.07-fold, respectively (Fig. 3E-G). These results suggested that CDR1as promoted the metastasis of cervical cancer.

### CDR1as promoted EMT in cervical cancer cells via binding with IGF2BP1

**Fig. 4:**
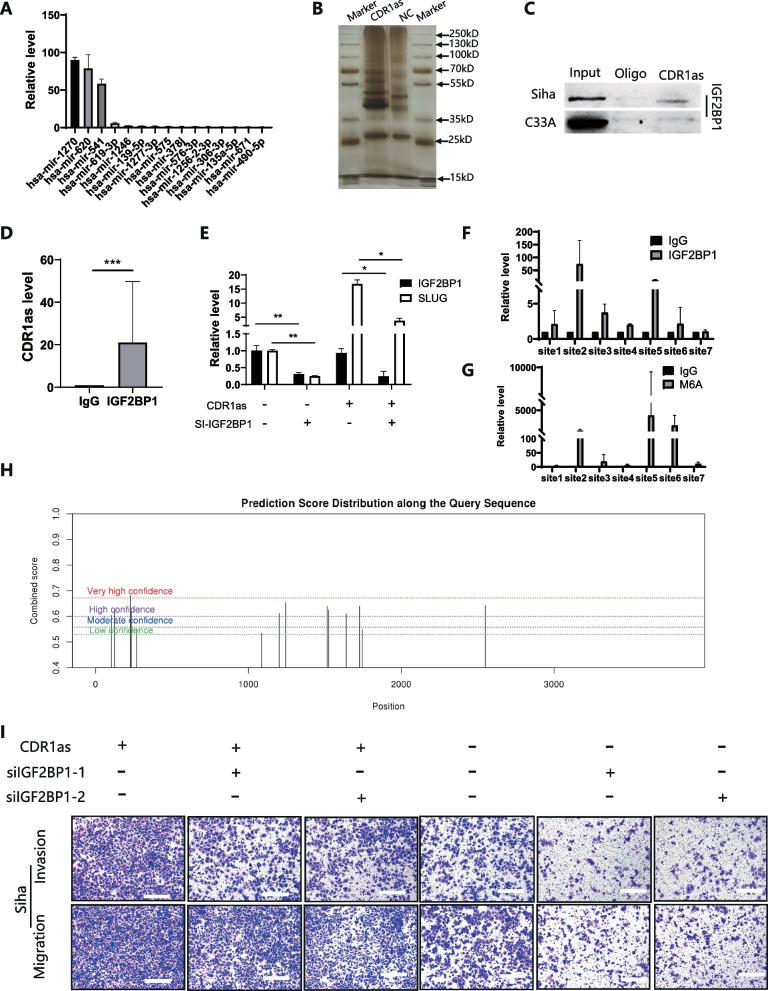
CDR1as promoted EMT in cervical cancer cells via binding with IGF2BP1. **A** qPCR was performed after pull-down using CDR1as probe; **B** Silver staining was performed after pull-down using CDR1as probe, a distinct band was discovered at 70 kD; **C** Western blot assay was performed after pull-down using CDR1as probe; **D** RIP assay using anti-IGF2BP1 shows an obvious enrichment of CDR1as; **E** qPCR was performed after IGF2BP1 was silenced; **F**,** G**,** H **Primers were designed for Slug’s M6A modification site, RIP assay using anti-m6A and anti- IGF2BP1 showed evident enrichment of Slug; **I** inhibition of IGF2BP1 reduced CDR1as-promoted metastatic and invasive potential

 As illustrated in Fig. 3J, after elevated CDR1as, cells adopted a spindle-shaped morphology while cells transfected with vector still exhibited a sharp margin on the vector; qPCR and Western blot suggested that the increase of CDR1as reduced E-cadherin expression and increased the expression of mesenchymal marker, including N-cadherin and vimentin (Fig. 3K, L), while *Slug* was upregulated the most. According to bioinformatic analysis, we overlapped the search results from www.circinteractome.irp.nia.nih.gov, www.mirdb.org, www.targetscan.org, and www.circbank.cn to identify potential binding between CDR1as and miRNAs. Twenty-five miRNAs were uncovered to bind with CDR1as. Among these miRNAs, miR-203 could regulate Slug expression, upon bio-informative prediction. However, CDR1as probe pull-down assay did not reveal Slug pathway-related miRNA (Fig. 4A), indicating that Slug activation was probably not being regulated by miRNA sponge.

Apart from competing endogenous RNAs (ceRNAs), several reports suggested that CDR1as was able to affect protein interaction. For example, it was found that CDR1as disrupted the p53/MDM2 complex to inhibit gliomagenesis [20]. In melanoma, CDR1as has been reported to unleash pro-metastatic functions of IGF2BP3, which shed new light onto the proteomics of CDR1as [10]. Hence, we performed RNA-pulldown to identify the potential proteins that could bind with CDR1as. It was found that there were several individual bands between 35kD to 70kD (Fig. 4B). Through bio-informative prediction from www.interactive.irp.nia.nih.gov, IGF2BPs were detected to interact with CDR1as, which was consistent with the previous mentioned research. Moreover, blots were discovered around 70 kD, which just coincided with the molecular weight of IGF2BPs. Then, we performed RIP and pull-down assays and found that IGF2BP 1/2/3 could bind with CDR1as. Specifically, only IGF2BP1 could be detected by CDR1as probe **(**Fig. 4C, D), indicating that IGF2BP1 could be a specific potential downstream target.

IGF2BP1 is well known for its implication in m6A regulation, and its major role was as an m6A reader. Especially, IGF2BP1 could recognize the modified site of m6A on mRNA, prevent the decay and maintain the stability of its targeted mRNA [21]. To explore whether IGF2BP1 affects CDR1as elevated Slug*, *we silenced IGF2BP1 in CDR1as overexpressed cells. It showed that reduced CDR1as induced Slug upregulation (Fig. 4E). Besides, RIP assays suggested that IGF2BP1 preferentially bound with m6A modified sites on the mRNA of Slug (Fig. 4F&H). Silencing IGF2BP1 also inhibited the metastatic and invasive potential promoted by CDR1as (Fig. 4I). Taken together, CDR1as bound specifically with IGF2BP1 to stabilize and elevate Slug expression.

### TGF-β signaling may activate EMT via CDR1as and m6A in cervical cancer cell

**Fig. 5:**
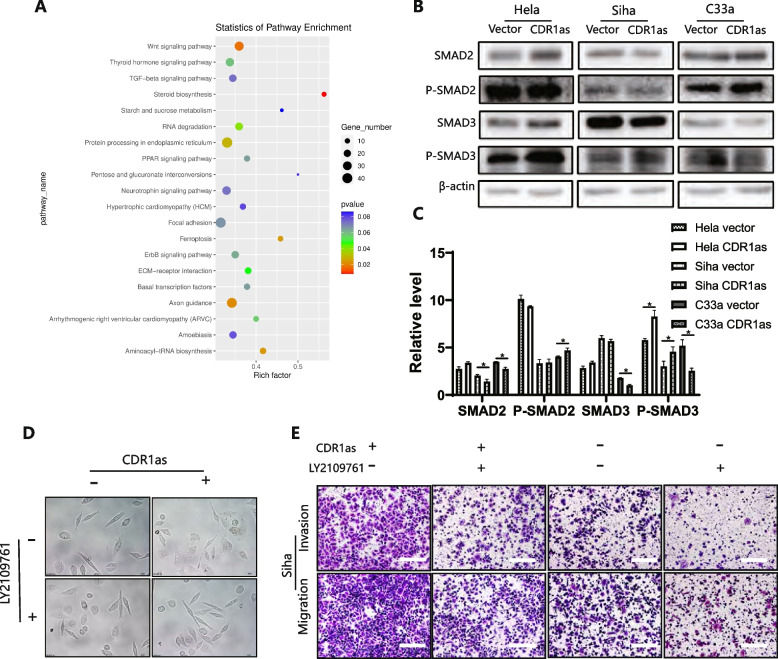
CDR1as activates TGF-β signaling and promotes EMT in cervical cancer cells; **A** Sanger sequencing shows that overexpressed CDR1as activated TGF-β signaling pathway; **B C** Western blot shows that P-SMAD2 and P-SMAD3 were upregulated after CDR1as was overexpressed; **D** Inhibition of TGF-β in CDR1as overexpressed cells reversed the EMT change by Wound healing; **E** Inhibition of TGF-β in CDR1as overexpressed cells reversed the EMT change by Transwell test

N6-methyladenosine is involved in almost all steps of RNA metabolism, which is an important part of transcriptional modification [22]. In conjunction with the preliminary verification of CDR1as functions and mechanisms, we then analyzed the levels of different mRNAs and m6A levels after the upregulation of CDR1as. KEGG and GO analysis indicated that CDR1as significantly activated the signal of TGF-β (Fig. 5A). Additionally, overexpressed CDR1as dramatically activated p-smad2 and p-smad3 by western blot (Fig. 5B-C). As expected, these EMT phenotypes were all reversed to the vector level when treating overexpressed CDR1as cells with LY2109761, which implied that CDR1as promoted EMT via activating TGF-β signaling (Fig. 5D). It was found that the number of penetrated cells were significantly decreased compared with the overexpressed CDR1as cells (Fig. 5E). These results suggested that CDR1as activated TGF-β signaling and EMT to promote metastasis in cervical cancer cells.

### CDR1as promoted progression of cervical cancer cells in vivo

**Fig. 6:**
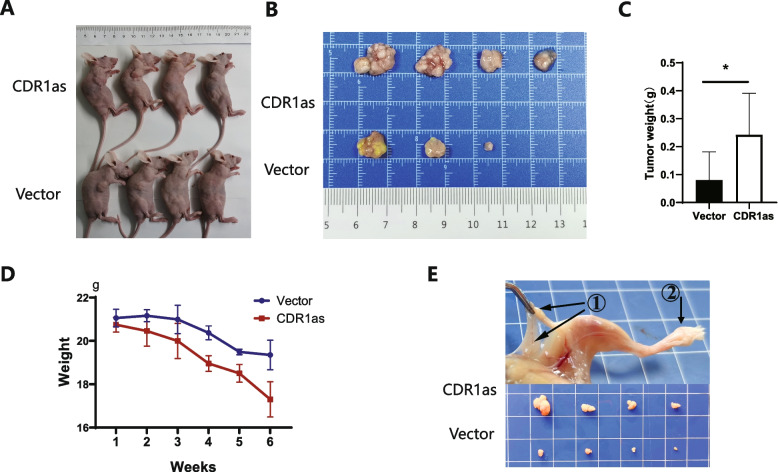
Over-expressed CDR1as promotes cervical cancer progression in vivo. **A, B, C, D** Overexpressed CDR1as of Siha was subcutaneously injected into mice, the mice were weighed once a week, tumors were harvested and weighted after 6 weeks; **E** Overexpressed CDR1as of Siha was injected into the foot pad of mice and popliteal lymph nodes were harvested after 6 weeks

**Fig. 7:**
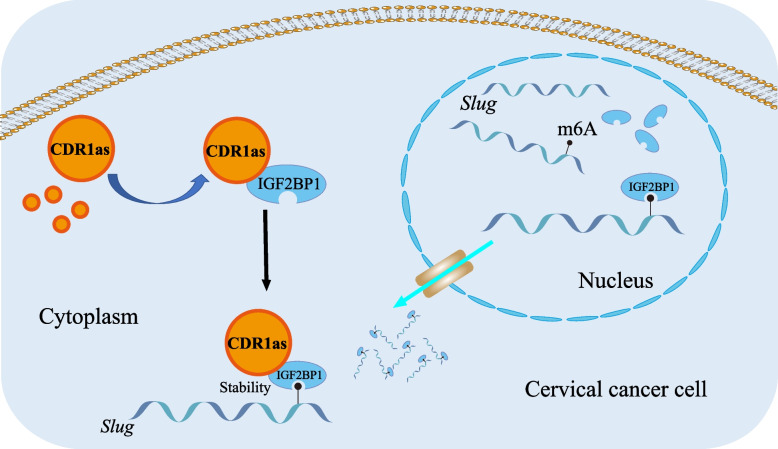
Cartoon for ‘TGF-β signaling may promote cervical cancer metastasis via CDR1as’ has been shown

We then evaluated the in vivo role of CDR1as on cervical cancer metastasis by injection of cervical cancer cells into the subcutaneous tissue and foot pad of mice. After 6 weeks of injection, the growth of primary tumors showed a difference between control group and CDR1as group. As for injected subcutaneously into anterior flank of nude mouse, the volume of tumor was increased compared to control group (Fig. 6A-B**)**, as well as a bigger tumor masses and reduced body weight when CDR1as was over-expressed (Fig. 6C-D**)**. As for injection of foot pad of mice, there was obvious swelling of Inguinal lymph nodes in CDR1as group (Fig. 6E). Cartoon for ‘TGF-β signaling may promote cervical cancer metastasis via CDR1as’ has been shown Fig. 7**.**

## Discussion

Most cervical cancer deaths are attributable to metastasis. Mechanisms responsible for metastasis are incompletely penetrated. TGF-β signaling has been reported to orchestrated an intricate signaling network to modulate tumorigenesis and cancer progression. Among multi-steps of TGF-β related metastasis in other cancers, R-SMAD/coSMAD complex was accumulated in the nucleus as transcription factors to promote EMT after TGF-β activation [23, 24]. Target genes including slug, snail, zeb1, zeb2, and twist were all known for promoting EMT [25]. To reveal the mechanism of TGF-β in metastatic spread would yield new insights of cervical cancer.

Epigenetic and transcriptional changes could drive metastatic processes. In our study, after treating cells with TGF-β molecular, metastasis was obviously promoted and the whole gene regulation and expression was reorganized. Among these changes, we focused on the circular RNA with regard to its extra-cellular stability and potential use as biomarker. Besides metastasis [15], CDR1as was also found to promote carcinogenesis [18] and cancer chemoresistance [26, 27]. In previous studies, CDR1as has been reported as a sponge for specific miRNAs, leading to regulation of downstream target mRNA expression and modulation of EMT processes. This mechanism has been commonly referred as a ceRNA network. However, we failed to identify a miRNA that could form a ceRNA network involved in the activation of Slug. Therefore, we speculate that other biomolecules may be involved in the regulation of Slug expression. 

 CircRNAs have been reported interacted with RNA binding proteins. CDR1as has been proved to interact with proteins such as IGF2BP3 and p53 [10, 20]. IGF2BPs belong to oncofetal proteins to regulate target mRNA transcripts in numerous cancers. In our study, after overexpression of CDR1as, the mRNA levels of IGF2BP1 did not demonstrate any change, while the protein levels of IGF2BP1 were significantly increased our data identify several IGF2BP3 targets that may contribute to this phenotype. Taken together, we hypothesize that IGF2BP1 is a key downstream effector of CDR1as.

 After overexpression of CDR1as, we also found one of TGF-β signaling target gene, Slug, was increased. Besides its role as an m6A reader, activated IGF2BP1 could bind with the promoter of multiple genes to promote translation. Silencing bp1 efficiently reduced the expression of TGF-β signaling-related factors. To the m6A modified site of Slug mRNA, this binding specifically improved the stability of Slug and prevented the decay of Slug.

## Conclusion

Our research addressed that effective CDR1as and m6A activate TGF-β signaling and promotes cervical cancer metastasis, which suggests potential therapeutic targets for cervical cancer.

## Data Availability

Not applicable.
